# Isoformic: a workflow for transcript-level RNA-seq interpretation

**DOI:** 10.1093/nargab/lqaf176

**Published:** 2025-12-03

**Authors:** Izabela Mamede, Lucio R Queiroz, Carlos Mata-Machado, Júlia Teixeira Rodrigues, Thomaz Luscher-Dias, Nayara E de Toledo, Paulo P Amaral, Luigi Marchionni, Gloria R Franco

**Affiliations:** Laboratory of Genetics Biochemistry, Departamento de Bioquímica e Imunologia, Universidade Federal de Minas Gerais, Belo Horizonte, Minas Gerais 31270-901, Brazil; D’Or Institute for Research and Education, São Paulo, SP, 01401-002, Brazil; Laboratory of Genetics Biochemistry, Departamento de Bioquímica e Imunologia, Universidade Federal de Minas Gerais, Belo Horizonte, Minas Gerais 31270-901, Brazil; Department of Pathology and Laboratory Medicine, Weill Cornell Medicine, New York, NY 10065, United States; Laboratory of Genetics Biochemistry, Departamento de Bioquímica e Imunologia, Universidade Federal de Minas Gerais, Belo Horizonte, Minas Gerais 31270-901, Brazil; Laboratory for Macromolecular Biophysics, Departamento de Bioquímica e Imunologia, Universidade Federal de Minas Gerais, Belo Horizonte, Minas Gerais 31270-901, Brazil; Laboratory of Genetics Biochemistry, Departamento de Bioquímica e Imunologia, Universidade Federal de Minas Gerais, Belo Horizonte, Minas Gerais 31270-901, Brazil; Laboratory of Genetics Biochemistry, Departamento de Bioquímica e Imunologia, Universidade Federal de Minas Gerais, Belo Horizonte, Minas Gerais 31270-901, Brazil; Instituto de Ensino e Pesquisa INSPER, São Paulo, SP 04546-042, Brazil; Department of Pathology and Laboratory Medicine, Weill Cornell Medicine, New York, NY 10065, United States; Laboratory of Genetics Biochemistry, Departamento de Bioquímica e Imunologia, Universidade Federal de Minas Gerais, Belo Horizonte, Minas Gerais 31270-901, Brazil

## Abstract

Transcriptome analysis is one of the bases of modern biology, yet it is typically performed at the gene level, ignoring the complexity of alternative splicing and differential transcription initiation/termination events. Over 95% of mammalian genes produce multiple transcripts, yet most RNA-seq analyses rely on short-read data, for which transcript-level interpretation remains challenging. Current tools suffer from low accuracy, inconsistency with annotations, and lack quick solutions for downstream biological interpretation. Here, we present Isoformic, a customizable R pipeline for transcript-level analysis of short-read RNA-seq data, available on GitHub. Isoformic processes differential expression results to detect genes with transcript-level changes, visualize exon-intron structures, and perform functional enrichment stratified by transcript type. Validated on diverse datasets, including preeclampsia, SARS-CoV-2 infection, and murine anxiety models, Isoformic reveals biologically relevant transcript variants and their possible phenotypic associations. Compatible with GENCODE reference transcriptome, Isoformic enhances the resolution of RNA-seq studies, enabling researchers to uncover the regulatory roles of alternative transcription events.

## Graphical Abstract

**Graphical Abstract:**
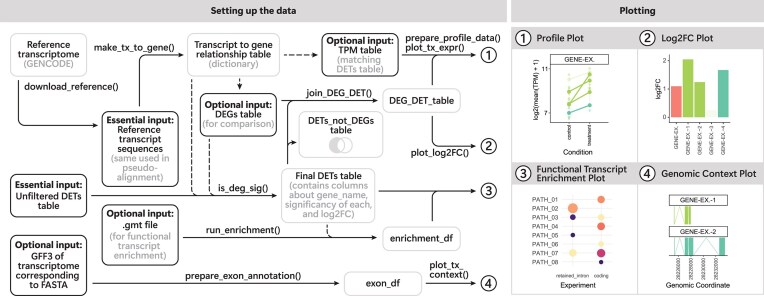

## Introduction

The human genome has <1.5% of its entirety annotated as protein-coding genes; however, >82% of the genome is transcribed [1]. Therefore, a significant portion of the produced RNA serves a regulatory function, like regulating splicing, genome, and chromatin integrity, and is not directed for translation [2, 3]. Protein-coding RNAs are annotated by the presence of open reading frames (ORFs). ORFs are generally defined in a nucleic acid sequence between the start codon and the stop codon of translation [4]. In eukaryotes, RNAs are processed during their synthesis, leading to different transcripts from the same gene region [5]. The three factors associated with transcript diversity are the transcription start point, the transcription endpoint, and splicing. The main diversity factor arises from different transcription start and end points [6], but the other factor in isoform diversity is splicing, an ultraconserved process since ancestral genomes [7], where introns are removed, and exons are retained. Alternative splicing occurs in over 95% of human multiexonic genes [8], and long non-coding RNAs are universally spliced [9]. This process can generate transcripts in which there is an ORF interruption due to the presence of introns, premature termination, or degradation markings, resulting mostly in non-protein-coding transcripts with a regulatory role [4, 10]. Even with all this variability in the transcriptome at the transcript level, RNA-seq studies are routinely analyzed at the gene level, where the expressions of transcripts spanning the same genomic region are combined to compute a total gene expression. This is due to the exponential increase in the complexity of analysis at the transcript level. While a transcriptome analyzed at the gene level returns a median of 400 differentially expressed genes, the same transcriptome analyzed at the transcript level could return over 3000 transcripts. Also, transcript-level analyses are highly dependent on annotation, and in organisms without well-annotated genomes or transcriptomes, this becomes impossible. Even for well-annotated transcriptomes, pipelines for transcript-level analysis generate many false positives, and using different pipelines yields results with little intersection that do not correspond to the annotation of the transcript type retrieved by long-read sequencing [11]. Recently, novel algorithms for analyzing transcript-level differential expression have emerged [12, 13]. These algorithms are designed to reduce dispersion in transcript counts and merge counts from highly similar transcripts that conventional methods struggle to distinguish [13, 14]. These approaches rely on reference transcriptomes, improving the reliability of transcript-level expression estimates. Nonetheless, interpretative challenges persist, with limited analytical frameworks available for interpreting transcript-level results. To address this need, we introduce Isoformic, a reproducible RNA-seq framework implemented as an R package. Isoformic was specifically developed for interpreting isoform-level expression in genomes with high-quality annotations of transcript classes and diversity. Isoformic facilitates the exploration of transcript differential expression and supports the extraction of biologically meaningful insights through diverse visualization methods. The package then incorporates gene and transcript annotations derived from a reference provided by the user or from the annotation files originally used during the quantification step. Our group has previously published results using Isoformic in SARS-CoV-2 infections [15], and the utility of Isoformic was validated using human datasets comparing pregnant women with preeclampsia and matched controls, as well as datasets derived from stressed mouse neurons.

## Materials and methods

Isoformic is an R package designed to leverage transcript counts and the associated Gibbs resamples generated by pseudo-alignment software such as Salmon. It provides a flexible framework for conducting differential transcript expression analyses and uses the resulting differential expression data and counts tables to generate intuitive visualizations highlighting differences among isoforms of the same gene.

Transcript-level quantification used Salmon [16] and recent GENCODE human or mice reference transcriptome annotations [6]. The index used in the pseudo-alignment was generated with k-mers of length 31 and resampled transcript counts using the Gibbs resampling algorithm, with the –dumpEqWeights (-d) parameter in Salmon. This method creates resampled counts using multiple iterations of Salmon in the same experiment. This is a crucial step for the subsequent analysis: (a) to collapse nearly similar transcripts with Terminus and (b) and to compute differential expression with swish or EdgeR [12, 13]. Terminus aims to group transcripts that have very similar intron and exon compositions, and thus, the pseudo-alignment of reads to these transcripts generates random distributions. Isoformic removes these clusters from the input for DE testing, which helps reduce spurious transcript-level calls caused by near-identical isoforms.

Differential expression analysis in the pipeline is performed using Salmon output processed with the swish function from the fishpond package [16], or as catchSalmon and glmQLFit (with legacy = FALSE) from edgeR v4 [12]. Both methods can directly incorporate the Gibbs resampling uncertainty form Salmon, whereas edgeR v4 offers efficient ways to do multiple group comparisons per run using the usual fit against a negative binomial distribution. Swish works in a case versus control manner using a non-parametric approach that does not rely on the negative binomial fit. Users can choose either method depending on their study design and preference. Isoformic is also compatible with long-read differential expression results if those were aligned to a reference, e.g. those generated by Oarfish [17] aligned to GENCODE, which can be imported in the same format as short-read outputs.

The threshold for identifying differential expression varied depending on the molecular type analyzed. For messenger RNA (mRNA) isoforms, the criteria were an absolute log_2_ fold-change ≥ 1 and an adjusted *P*-value (*q*-value) < .05, while for lncRNA isoforms, a less stringent log_2_ fold-change ≥ 0.5 with adjusted *P*-value (*q*-value) < .05 was applied. This adjustment reflects previous findings showing that lncRNAs have lower expression levels compared to protein-coding genes [18], particularly when analyzing individual isoforms. Thus, applying a stricter threshold of log_2_ fold-change ≥ 1 would risk omitting biologically relevant lncRNA isoforms from the results.

## Results

### Input data formatting

Isoformic requires four key inputs: (i) differential transcript expression, (ii) differential gene expression, (iii) gene counts, and (iv) transcript counts. If users employed a GENCODE reference during their analysis, they could conveniently obtain the corresponding annotation directly within Isoformic using the download_reference function. This function enables the retrieval of transcript metadata annotations (in GTF and GFF formats) and associated sequences (in FASTA format) directly from the GENCODE database. These annotations are subsequently structured into a comprehensive dictionary containing exon-level and transcript-type information. The dictionary is generated via the make_tx_to_gene function, which parses GENCODE FASTA file headers, GTF, or GFF files into structured data columns, which are then integrated with the differential expression data. Isoformic is particularly optimized for analyses involving human and mouse data, as these two organisms currently possess the most robust and extensively curated transcript-level annotations [6]. Additionally, Isoformic generates a specialized table highlighting transcripts with differential expression but whose corresponding genes do not show differential expression. This information helps identify potential isoform switches or differential transcript-level regulatory events.

### Pairwise comparison of transcript abundance: case versus control

**Figure 1.**
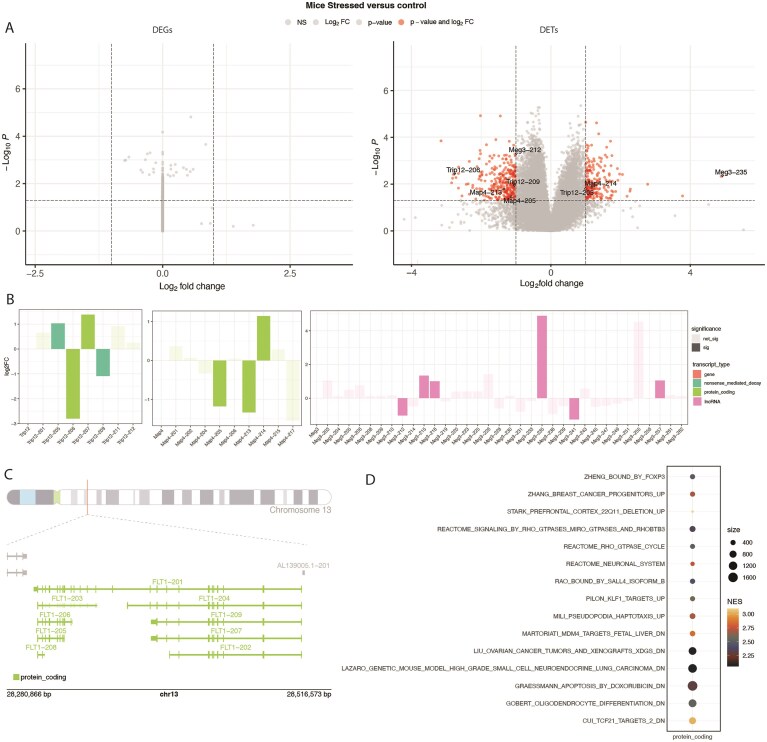
Isoformic application in mice neuron experiment. (**A**) Volcano plots of the differential expression of stressed versus not stressed mice at gene (left) and at transcript level (right). In red, genes or transcripts that pass on the cutoff values of *P*-value < .05 and log_2_ foldchange > 1. (**B**) Log_2_ fold-change plot of two different genes and their isoform expression on stressed versus control mice. Transcript types are represented as bar colors, and non-significant transcripts are transparent when compared to the significant ones. (**C**) Genomic context plot of the FLT1 isoforms presenting the dataset. Exons are presented as rectangles, and introns as lines. (**D**) Functional enrichment of the differentially expressed protein-coding isoforms.

Isoformic provides two visualization outputs, a line graph and a bar chart (Fig. 1A and B), enabling users to effectively compare isoform abundance between conditions. The bar chart displays the log_2_ fold-change for each isoform and its corresponding gene, along with statistical significance. The line graph illustrates the differences in transcripts per million between groups, including arrows representing the standard deviation for each sample. Together, these visualizations help identify molecular phenotypes driven by isoform-level differences, which might be missed by gene-centric analyses. Users may additionally supply multiple case–control comparisons within the same DE table, and Isoformic functions can facet the visualization outputs accordingly, as these are implemented in ggplot2.

### Exon and intron composition differences

The second comparison performed by Isoformic involves analyzing intron-exon compositions of transcripts from the same gene. Typically, this type of visualization can only be achieved through genome browsers such as ENSEMBL and UCSC [19, 20], which often require additional manual adjustments to convert browser screenshots into publication-ready figures. Alternatively, visualizations can be generated from alignment outputs in BAM or SAM formats [21], but this approach significantly increases both processing time and file sizes. To streamline this process, we implemented a more direct method, utilizing a GenomicRanges object [22] created from exon coordinates obtained using the prepare_exon_annotation function. The resulting exon table is then passed to the plot_tx_context function, generating a genomic context visualization (Fig. 1C). In this plot, transcript models are ordered according to genomic coordinates, ensuring that sense-strand transcripts begin on the left and antisense-strand transcripts on the right (schematic representation of this in Supplementary Fig. S1). This was made using an implementation of the plotGardener package [23].

### Functional enrichment at the transcript level

One of the major limitations of transcript-level analysis is that it is often difficult to extract biologically relevant information from such a large amount of data. The next step for gene-level differential expression would be functional enrichment or assigning genes to metabolic pathways they may be regulating. Unfortunately, there are no comprehensive datasets for the pathways that transcripts may be directly regulating, and gene-level analyses typically miss differences between those transcripts that can produce proteins (protein_coding) from canonical translation pathways and those that cannot. To address this problem, we developed a method to expand the databases used (which are typically deposited in GMT format) for transcript information, and then separately enrich each type of transcript (retained-intron, nonsense-mediated decay, CDS_undefined, and protein_coding). Isoformic achieves this by projecting each transcript’s parent gene annotation onto the corresponding transcript and then stratifying enrichment by transcript biotype. We note that this approach inherits the limitations of gene-level annotations and assumes that protein-coding transcripts mirror their main genes and non-protein-coding are the opposite of it. Nonetheless, this extension enables a first step toward uncovering functional differences between protein-coding and non-protein-coding isoforms. We also grouped the three main isoform types that are results of alternative mRNA processing into a non-protein-coding isoform enrichment, to be able to detect what these do in direct opposition of the coding enrichment. This enrichment distinction between protein-coding and non-protein-coding was used before to show how SARS-CoV-2 infection influences coding and non-coding transcripts [16].

Isoformic provides the functionality to convert a user-inputted GMT file containing any gene-level database, which can come from the Gene Ontology, REACTOME, among others [24, 25], into a transcript database. The package then uses the run_enrichment function, which modifies this user’s GMT file to a transcript-level database separated by type and enriches the DET table using the fgsea package [26]. The result will be a functional enrichment table separated by transcript categories, and with this table, the user can subsequently generate an annotated dot that separate enrichments in the categories and allows visualization of phenotypes possibly associated with each transcript category, enabling the user to draw conclusions from categories that are being enriched by protein-coding transcripts versus those that are not.

### Application to mouse neuron dataset

We applied Isoformic to the analysis of mouse neurons under stress conditions. These stimuli activate the body’s stress-response system, ideally prompting adaptive reactions. However, prolonged exposure to stress, whether acute or chronic, can lead to dysregulation of this system, contributing to the development of conditions like anxiety, depression, cardiovascular diseases, and metabolic syndrome [27]. Analysis of RNA sequencing data in psychiatric disorders suggests alternative splicing as a crucial molecular mechanism underlying conditions such as autism spectrum disorder, schizophrenia, and bipolar disorder [28], [29]. Given the observed splicing changes in stress-related studies, it’s plausible that alternative splicing plays a pivotal role in the development of both psychiatric and behavioral disorders.

To evaluate Isoformic, we chose the PRJNA984605 BioProject, which examined chronic stress in mice [28]. RNA was extracted from the anterodorsal division of the bed nucleus of the stria terminalis, a brain region closely linked to stress and anxiety. This study included both male and female mice. Notably, when comparing same-sex stress-induced and control mice, the authors found no differentially expressed genes [28]. When we executed our pipeline, we were able to find over 200 differentially expressed transcripts using stressed versus control mice, regardless of sex (Fig. 1A). Isoformic was also able to distinguish different isoforms for important neuroregulatory genes such as Trip12 (thyroid hormone receptor interactor 12) and Map4 (microtubule-associated protein 4) (Fig. 1B) and detect different isoforms of lncRNA genes like Meg3. The coding isoforms of Trip12 that are present are upregulated and downregulated; Trip-206 has an extra exon affecting the isoform’s termination point.

### Future extensions

In further versions, we plan to integrate all the inputs needed for the package into a single S4 object to streamline the analysis process. This consolidation will simplify data handling and reduce the potential for user error, making the workflow more efficient and user-friendly. The S4 structure will also facilitate more complex experimental designs, such as time series or multifactor studies, by allowing metadata, quantifications, and differential expression results to be stored and accessed in a coordinated manner. We also aim to provide an end-to-end workflow that guides users on processing raw sequencing reads directly into Isoformic-compatible files. We recognize the importance of extending support to other model and non-model species with available genome annotations, providing mechanisms to use custom transcript classes.

## Supplementary Material

lqaf176_Supplemental_File

## Data Availability

No new data were generated in this project, and all test data are publicly available in SRA (PRJNA623568 and PRJNA984605) and GitHub (https://github.com/luciorq/isoformic). We have archived the exact version of Isoformic used to produce the results on the Zenodo record (DOI: 10.5281/zenodo.17267590; https://doi.org/10.5281/zenodo.17267590). The package documentation also includes three vignettes: (i) one using a small, pre-packaged dataset to demonstrate how to process your data before Isoformic, (ii) one showing Isoformic’s core functions, and (iii) another showing how Isoformic can be applied to long-read differential expression results (e.g. from Oarfish aligned to GENCODE).
