# Supplementary material for: Isoformic: a workflow for transcript-level RNA-seq interpretation
Source: NAR Genom Bioinform. 2025 Dec 3;7(4):lqaf176. doi: 10.1093/nargab/lqaf176 (PMC12673842; doi:10.1093/nargab/lqaf176)
Supplement: lqaf176_Supplemental_File [file lqaf176_supplemental_file.pdf]

Example of Context Plot

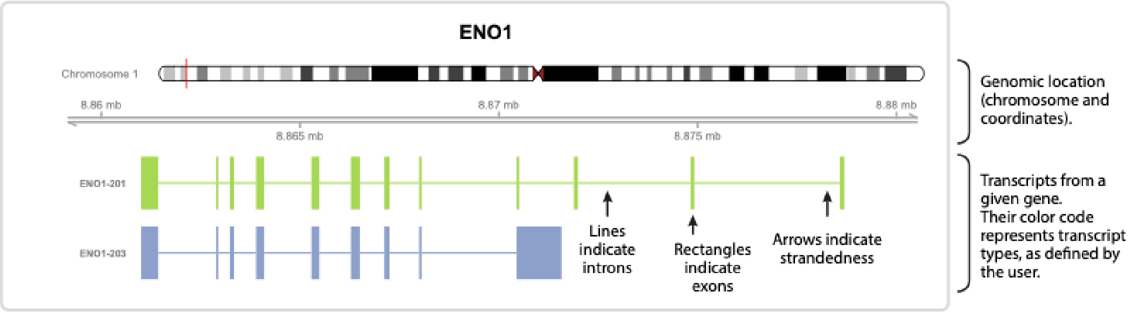

Figure S1. Example of a genomic context plot.

Each panel represents the genomic locus of a selected gene, showing its genomic position, gene structure, and isoform-specific features derived from transcript-level quantification. The top schematic (Chromosome 1 ideogram) shows the chromosomal location of the locus (here: 8.85–8.89 Mb on chr1). Below, the horizontal scale bar indicates genomic coordinates (in Mb), and the rectangles correspond to exons of individual transcript isoforms aligned to the genome (introns are shown as connecting lines). Each isoform is drawn using coordinates from the reference annotation and colored by biotype (e.g., protein-coding in green and lncRNA in blue). No alignment data are used directly; exon coordinates are derived from the annotation, and expression information is overlaid from quantified transcripts.
